# Prelaryngeal and/or pretracheal lymph node metastasis could help to identify papillary thyroid carcinoma with intermediate risk from unilateral lobe cT1-2N0 papillary thyroid carcinoma

**DOI:** 10.3389/fendo.2023.1156664

**Published:** 2023-04-14

**Authors:** Bin Wang, Chun-Rong Zhu, Yuan Fei, Hong Liu, Xin-Min Yao, Jian Wu

**Affiliations:** ^1^ Center of Breast and Thyroid Surgery, Department of General Surgery, The Third People’s Hospital of Chengdu, Chengdu, Sichuan, China; ^2^ Department of Oncology Ward 2, The Third People’s Hospital of Chengdu, Chengdu, Sichuan, China

**Keywords:** pretracheal lymph node, prelaryngeal lymph node, paratracheal lymph node, papillary thyroid carcinoma, intermediate risk

## Abstract

**Objective:**

The study aims to explore the possibility of prelaryngeal and/or pretracheal lymph node metastasis in identifying papillary thyroid carcinoma with more than 5 metastatic central lymph nodes from unilateral lobe cT1-2N0 papillary thyroid carcinoma.

**Methods:**

A retrospective analysis was conducted on patients who underwent the initial thyroid surgery for unilateral lobe cT1-2N0 PTC in a single tertiary center between July 2018 to December 2022. Multivariable binary logistic regression analysis was used to identify risk factors for unilateral lobe cT1-2N0 papillary thyroid carcinoma with more than 5 metastatic central lymph nodes.

**Results:**

A total of 737 patients were included in the study and 399 patients were confirmed to suffer from occult central lymph node metastasis. The larger size of the largest diameter of tumor (> 1cm; OR = 3.3, 95%CI 1.6 – 6.83; p = 0.001), pretracheal lymph node metastasis (OR = 5.91, 95%CI 2.73 – 12.77; p < 0.001), prelaryngeal lymph node metastasis (OR = 3.74, 95%CI 1.73 – 8.1; p = 0.001), ipsilateral paratracheal lymph node metastasis (OR = 12.22, 95%CI 3.43 – 43.48; p < 0.001), and contralateral paratracheal lymph node metastasis (OR = 7.68, 95%CI 3.86 – 15.3; p < 0.001) were confirmed to be risk factors for unilateral lobe cT1-2N0 PTC with more than 5 metastatic central lymph nodes. When more than two metastatic prelaryngeal and/or pretracheal lymph nodes occurred, the incidence of more than 5 metastatic central lymph nodes was 71.2%.

**Conclusion:**

Prelaryngeal and/or pretracheal lymph node metastasis could help to identify papillary thyroid carcinoma with more than 5 metastatic central lymph nodes from unilateral lobe cT1-2N0 papillary thyroid carcinoma. When more than two metastatic pretracheal and/or prelaryngeal lymph nodes occurred, total thyroidectomy and ipsilateral central lymph node dissection should be performed and contralateral paratracheal lymph node dissection might be also necessary.

## Introduction

Although central lymph node metastasis occurred in 36.4%~64.7% of clinically node-negative papillary thyroid carcinoma (cN0 PTC) (1–3), the idea that prophylactic central neck dissection should not be routinely performed for small (T1 or T2), noninvasive, cN0 PTC is strongly recommended by the 2015 American Thyroid Association Management Guidelines for Adult Patients with Thyroid Nodules and Differentiated Thyroid Cancer (2015 ATA Guidelines) with moderate-quality evidence (4). The reason for the recommendation was that the 2015 ATA Guidelines concluded that prophylactic dissection does not have an improvement in long-term patient outcome with increasing the likelihood of temporary morbidity and that the effect of the upgrade stage from cN0 to pN1 on overall survival is small (4). While, according to the 2015 ATA Initial Risk Stratification System, it was regarded as the intermediate risk that more than 5 metastatic central lymph nodes with all involved lymph nodes <3 cm in largest dimension (4). And patients with intermediate-risk level PTC should be considered to accept radioactive iodine adjuvant therapy, which means total thyroidectomy is necessary (4). At present, there are no guidelines, consensus, or indicators for total thyroidectomy and central neck dissection for cT1-2N0 PTC with potential intermediate risk.

The central lymph node consists of the prelaryngeal lymph node, pretracheal lymph node, and paratracheal (or trachea-esophageal groove) lymph node (5, 6). In recent years, several studies explored the significance of prelaryngeal lymph node metastasis in PTC and found that prelaryngeal lymph node metastasis was positively associated with paratracheal and lateral lymph node metastasis (3, 7–10). A similar result was obtained between pretracheal lymph node metastasis and paratracheal lymph node metastasis (6, 11, 12). However, some aforementioned studies included isthmic PTC and/or bilateral lobe PTC, which might increase the risk of lymph node metastasis and then the relationship between prelaryngeal/pretracheal lymph node metastasis and paratracheal lymph node metastasis might be affected.

Here, we conducted the retrospective study to explore the possibility of prelaryngeal and/or pretracheal lymph node metastasis in identifying PTC with more than 5 metastatic central lymph nodes from unilateral lobe cT1-2N0 PTC, and assess the association of prelaryngeal and/or pretracheal lymph node metastasis with paratracheal lymph node metastasis, and identify risk factors for unilateral lobe cT1-2N0 PTC with more than 5 metastatic central lymph nodes.

## Patients and methods

### Patients

The study began with a review of patients who underwent thyroid surgery for PTC at our institution from July 2018 to December 2022. Patients who underwent the initial thyroid surgery for unilateral lobe cT1-2N0 PTC were considered for inclusion. Patients with bilateral lobe PTC or isthmic PTC confirmed by postoperative pathological reports were excluded. The study was approved by the Medical Ethics Committee of The Third People’s Hospital of Chengdu. And informed consent about the application of clinical data to medical research was routinely obtained from all the subjects before they were discharged.

### Indications for surgery and surgical procedure

Total thyroidectomy was performed for the following indications: 1) the largest diameter of the tumor was more than 1cm; 2) the preoperative image discovered thyroid nodule in the contralateral lobe; 3) capsular invasion was confirmed by intraoperative frozen pathology; 4) prelaryngeal and/or pretracheal lymph node metastasis was confirmed by intraoperative frozen pathology. Otherwise, lobe thyroidectomy was performed. Ipsilateral central lymph node (including prelaryngeal lymph node, pretracheal lymph node, and ipsilateral paratracheal lymph node) dissection was routinely performed. Contralateral paratracheal lymph node dissection was performed when capsular invasion and/or prelaryngeal and/or pretracheal lymph node metastasis was confirmed by intraoperative frozen pathology.

Two professional thyroid surgeons (Wu J and Yao X) performed all surgeries. The surgical procedures are conducted as follows. After anesthesia, the patient was adjusted to hyperextension of the head. A transverse incision located at 1cm beyond the suprasternal fossa was the surgical entrance. The flap with platysma muscle was then dissociated and it bordered superiorly by the thyroid cartilage, inferiorly by the sternum, and laterally on each side by the sternocleidomastoid muscle. After the strap muscles were separated *via* linea alba cervicalis, the space between the thyroid with central tissue and strap muscles was opened. Following that, the prelaryngeal and pretracheal tissues were routinely resected and examined by intraoperative frozen pathology. After that, thyroidectomy and paratracheal lymph node dissection were performed in sequence. The resected thyroid was also routinely examined by intraoperative frozen pathology.

### Data collection

The following data were collected: demographic characteristics, comorbidities, tumor characteristics, details of surgical extent, and the number of lymph nodes and metastatic lymph nodes in each subgroup of the central zone.

### Statistical analysis

All the statistical analyses were performed using SPSS version 23.0 software (SPSS Inc, Chicago, IL). Continuous data were expressed as mean ± standard deviation (SD) and analyzed using Student’s t-test or Mann–Whitney test. Categorical data were shown as absolute numbers and analyzed using Pearson’s Chi-square test or Fisher’s exact test. These variables with potentially statistically significant in univariate analysis (p < 0.1) were included in the multivariable binary logistic regression analysis. Statistical significance was set at P < 0.05.

## Result

A total of 737 patients were included in the study and 399 patients were confirmed to suffer from occult central lymph node metastasis. Among them, 198, 58, 350, and 81 patients suffered from pretracheal lymph node metastasis, prelaryngeal lymph node metastasis, ipsilateral paratracheal lymph node metastasis, and contralateral paratracheal lymph node metastasis, respectively. Prelaryngeal lymph node metastasis and pretracheal lymph node metastasis simultaneously occurred in forty-one patients. Among these patients without prelaryngeal lymph node metastasis, 436 had no lymph node in the prelaryngeal tissue. It happened to 47 patients that there was no lymph node in the pretracheal tissue. Three hundred and thirty-three cases suffered from left-lobe PTC. Among them, 43, 139, and 151 underwent left lobe thyroidectomy plus left central lymph node dissection, total thyroidectomy plus left central lymph node dissection, and total thyroidectomy plus bilateral central lymph node dissection, respectively.

According to the univariate analysis, there were no significant differences in gender, hyperthyroidism, hypothyroidism, and Hashimoto’s Thyroiditis between patients with pretracheal lymph node metastasis and patients without pretracheal lymph node metastasis (**Table 1**). The age, hypertension, diabetes, nodular goiter, multifocal tumors, capsular invasion, the largest diameter of tumor, and tumor location were associated with pretracheal lymph node metastasis (P = 0.001, P =0.049, P =0.031, P = 0.023, P = 0.008, P < 0.001, P < 0.001, and P =0.014, respectively; **Table 1**). It was significantly different in the incidences of prelaryngeal lymph node metastasis (P < 0.001, **Table 1**), ipsilateral paratracheal lymph node metastasis (P < 0.001, **Table 1**), contralateral paratracheal lymph node metastasis (P < 0.001, **Table 1**), and more than 5 metastatic central lymph nodes (P < 0.001, **Table 1**) between patients with and without pretracheal lymph node metastasis. The multivariate analysis indicated that young (< 55y; OR = 0.38, 95%CI 0.23 – 0.64; p < 0.001; **Table 1**), unifocal tumors (OR = 0.51, 95%CI 0.21 – 0.98; p = 0.044; **Table 1**), capsular invasion (OR = 2.7, 95%CI 1.88 – 3.87; p < 0.001; **Table 1**), the larger size of the largest diameter of tumor (> 1cm; OR = 2.45, 95%CI 1.71 – 3.52; p < 0.001; **Table 1**), and non-upper lesion (OR = 0.59, 95%CI 0.39 – 0.89; p = 0.013; **Table 1**) were independent risk factors for pretracheal lymph node metastasis.

**Table 1:** The risk factors for pretracheal lymph node metastasis.

Variables	Univariate Analysis			Multivariate Analysis (G=761.335, P<0.001)		
	PT-LN(+) n=198	PT-LN(-) n=539	P	OR	95%CI	P
Gender(F/M)	134/64	397/142	0.109			
Age (years)	38.6 ± 11.8	45.6 ± 12.2	<0.001			
≥55	22	117	0.001	0.38	0.23 — 0.64	<0.001
<55	176	422				
Hypertension	13	62	0.049			
Diabetes	1	18	0.031			
Hyperthyroidism	5	18	0.573			
Hypothyroidism	2	9	0.755			
Hashimoto’s Thyroiditis	64	147	0.179			
Nodular goiter	90	296	0.023			
Multifocal tumors	12	70	0.008	0.51	0.26 — 0.98	0.044
Capsular invasion	109	144	<0.001	2.7	1.88 — 3.87	<0.001
Largest tumor size (mm)	14.6 ± 7.4	10.9 ± 6.8	<0.001			
>10(%)	126 (38.3)	203 (61.7)	<0.001	2.45	1.71 — 3.52	<0.001
≤10(%)	72 (17.6)	336 (82.4)				
Tumor location (upper/middle/lower)	42/87/69	155/238/146	0.014	0.59	0.39 — 0.89	0.013
Surgical extent			<0.001			
LT+UCND	0	91				
TT+UCND	0	301				
TT+BCND	198	147				
PL-LNM(%)	41 (70.7)	17 (29.3)	<0.001			
Ipa-LNM(%)	160 (45.7)	190 (54.3)	<0.001			
Cpa-LNM(%)	69 (85.2)	12 (14.8)	<0.001			
Total number of metastatic lymph nodes	5.3 ± 3.9	0.9 ± 1.6	<0.001			
>5(%)	73 (86.9)	11 (13.1)	<0.001			
≤5(%)	125 (19.1)	528 (80.9)				

PT-LN, pretracheal lymph node; F, female; M, male; LT, lobe thyroidectomy; TT, total thyroidectomy; UCND, unilateral central lymph node dissection; BCND, bilateral central lymph node dissection; PL-LNM, prelaryngeal lymph node metastasis; Ipa-LNM, ipsilateral paratracheal lymph node metastasis; Cpa-LNM, contralateral paratracheal lymph node metastasis.

The univariate analysis suggested that gender, capsular invasion, and the largest diameter of the tumor might be related to prelaryngeal lymph node metastasis (**Table 2**). The multivariate analysis further confirmed that male (OR = 0.4, 95%CI 0.23 – 0.7; p = 0.001; **Table 2**), capsular invasion (OR = 2.81, 95%CI 1.57 – 5.03; p = 0.001; **Table 2**), and larger size of the largest diameter of tumor (> 1cm; OR = 2.11, 95%CI 1.15 – 3.88; p = 0.016; **Table 2**) were independent risk factors for prelaryngeal lymph node metastasis. There were also significant differences in the incidences of pretracheal lymph node metastasis (P < 0.001, **Table 2**), ipsilateral paratracheal lymph node metastasis (P < 0.001, **Table 2**), contralateral paratracheal lymph node metastasis (P < 0.001, **Table 2**), and more than 5 metastatic central lymph nodes (P < 0.001, **Table 2**) between patients with and without prelaryngeal lymph node metastasis.

**Table 2:** The risk factors for prelaryngeal lymph node metastasis.

Variables	Univariate Analysis			Multivariate Analysis (G=370.567, P<0.001)		
	PL-LN(+) n=58	PL-LN (-) n=679	P	OR	95%CI	P
Gender (F/M)	31/27	500/179	0.001	0.4	0.23 — 0.7	0.001
Age (years)	39.9 ± 13.1	44.1 ± 12.4	0.13			
≥55	9	130	0.498			
<55	49	549				
Hypertension	5	70	0.683			
Diabetes	0	19	0.39			
Hyperthyroidism	2	21	0.701			
Hypothyroidism	0	11	0.68			
Hashimoto’s Thyroiditis	19	192	0.469			
Nodular goiter	32	354	0.657			
Multifocal tumors	6	76	0.844			
Capsular invasion	35	218	<0.001	2.81	1.57 — 5.03	0.001
Largest tumor size (mm)	16.2 ± 8.8	11.6 ± 6.9	<0.001			
>10 (%)	40 (12.2)	289 (87.8)	<0.001	2.11	1.15 — 3.88	0.016
≤10 (%)	18 (4.4)	390 (95.6)				
Tumor location (upper/middle/lower)	14/29/15	183/296/200	0.931			
Surgical extent			<0.001			
LT+UCND	0	91				
TT+UCND	0	301				
TT+BCND	58	287				
PT-LNM (%)	41 (20.7)	157 (79.3)	<0.001			
Ipa-LNM (%)	47 (13.4)	303 (86.6)	<0.001			
Cpa-LNM (%)	28 (34.6)	53 (65.4)	<0.001			
Total number of metastatic lymph nodes	7.3 ± 5.2	1.6 ± 2.4	<0.001			
>5 (%)	30 (35.7)	54 (65.3)	<0.001			
≤5 (%)	28 (4.3)	625 (95.7)				

PL-LN, prelaryngeal lymph node; F, female; M, male; LT, lobe thyroidectomy; TT, total thyroidectomy; UCND, unilateral central lymph node dissection; BCND, bilateral central lymph node dissection; PT-LNM, pretracheal lymph node metastasis; Ipa-LNM, ipsilateral paratracheal lymph node metastasis; Cpa-LNM, contralateral paratracheal lymph node metastasis.

As shown in **Table 3**, gender, age, diabetes, capsular invasion, the largest diameter of tumor, pretracheal lymph node metastasis, and prelaryngeal lymph node metastasis might be associated with ipsilateral paratracheal lymph node metastasis. It was confirmed that male (OR = 0.57, 95%CI 0.4 – 0.82; p = 0.002; **Table 3**), the larger size of the largest diameter of tumor (> 1cm; OR = 1.8, 95%CI 1.3 – 2.5; p < 0.001; **Table 3**), pretracheal lymph node metastasis (OR = 6.23, 95%CI 4.13 – 9.4; p < 0.001; **Table 3**), and prelaryngeal lymph node metastasis (OR = 2.22, 95%CI 1.05 – 4.66; p = 0.036; **Table 3**) were independent risk factors for ipsilateral paratracheal lymph node metastasis by the multivariate analysis. The incidences of contralateral paratracheal lymph node metastasis (20.3% vs 2.6%, p < 0.001; **Table 3**) and more than 5 metastatic central lymph nodes (23.1% vs 0.8%, p < 0.001; **Table 3**) were higher in patients with ipsilateral paratracheal lymph node metastasis than that in patients without ipsilateral paratracheal lymph node metastasis.

**Table 3:** The risk factors for ipsilateral paratracheal lymph node metastasis.

Variables	Univariate Analysis			Multivariate Analysis (G=862.978, P<0.001)		
	Ipa-LN(+) n=350	Ipa-LN (-) n=387	P	OR	95%CI	P
Gender (F/M)	229/121	302/85	<0.001	0.57	0.4 — 0.82	0.002
Age (years)	41.5 ± 12.0	45.8 ± 12.5	<0.001			
≥55	53	86	0.014			
<55	297	301				
Hypertension	32	43	0.377			
Diabetes	4	15	0.019			
Hyperthyroidism	9	14	0.415			
Hypothyroidism	6	5	0.637			
Hashimoto’s Thyroiditis	95	116	0.396			
Nodular goiter	179	207	0.524			
Multifocal tumors	37	45	0.649			
Capsular invasion	143	110	<0.001			
Largest tumor size (mm)	13.7 ± 7.7	10.3 ± 6.3	<0.001			
>10 (%)	197 (59.9)	132 (40.1)	<0.001	1.8	1.3 — 2.5	<0.001
≤10 (%)	153 (37.5)	255 (62.5)				
Tumor location (upper/middle/lower)	87/150/113	110/175/102	0.084			
Surgical extent			<0.001			
LT+UCND	22	69				
TT+UCND	105	196				
TT+BCND	223	122				
PT-LNM (%)	160 (80.8)	38 (19.2)	<0.001	6.23	4.13 — 9.4	<0.001
PL-LNM (%)	47 (81.0)	11 (19.0)	<0.001	2.22	1.05 — 4.66	0.036
Cpa-LNM (%)	71 (87.7)	10 (12.3)	<0.001			
Total number of metastatic lymph nodes	4.0 ± 3.5	0.3 ± 0.9	<0.001			
>5 (%)	81 (96.4)	3 (3.6)	<0.001			
≤5 (%)	269 (41.2)	384 (58.8)				

Ipa-LN, ipsilateral paratracheal lymph node; F, female; M, male; LT, lobe thyroidectomy; TT, total thyroidectomy; UCND, unilateral central lymph node dissection; BCND, bilateral central lymph node dissection; PT-LNM, pretracheal lymph node metastasis; PL-LNM, prelaryngeal lymph node metastasis; Cpa-LNM, contralateral paratracheal lymph node metastasis.

**Table 4** showed the risk factors for contralateral paratracheal lymph node metastasis. Although age, capsular invasion, the largest diameter of the tumor, tumor location, pretracheal lymph node metastasis, prelaryngeal lymph node metastasis, and ipsilateral paratracheal lymph node metastasis were discovered association with contralateral paratracheal lymph node metastasis by the univariate analysis, the multivariate analysis demonstrated that larger size of the largest diameter of tumor (> 1cm; OR = 2.64, 95%CI 1.43 – 4.87; p = 0.002; **Table 4**), pretracheal lymph node metastasis (OR = 11.2, 95%CI 5.62 – 22.32; p < 0.001; **Table 4**), prelaryngeal lymph node metastasis (OR = 4.3, 95%CI 2.16 – 8.55; p < 0.001; **Table 4**), and ipsilateral paratracheal lymph node metastasis (OR = 2.84, 95%CI 1.32 – 6.07; p = 0.007; **Table 4**) were independent risk factors for contralateral paratracheal lymph node metastasis. There were more metastatic lymph nodes in patients with contralateral paratracheal lymph node metastasis (**Table 4**).

**Table 4:** The risk factors for contralateral paratracheal lymph node metastasis.

Variables	Univariate Analysis			Multivariate Analysis (G=329.058, P<0.001)		
	Cpa-LN(+) n=81	Cpa-LN(-) n=656	P	OR	95%CI	P
Gender (F/M)	51/30	480/176	0.053			
Age (years)	38.5 ± 13.1	44.4 ± 12.2	<0.001			
≥55	9	130	0.059			
<55	72	526				
Hypertension	9	66	0.768			
Diabetes	1	18	0.711			
Hyperthyroidism	4	19	0.306			
Hypothyroidism	1	10	>0.99			
Hashimoto’s Thyroiditis	22	189	0.757			
Nodular goiter	42	344	0.92			
Multifocal tumors	11	71	0.457			
Capsular invasion	48	205	<0.001			
Largest tumor size (mm)	16.6 ± 8.6	11.3 ± 6.8	<0.001			
>10 (%)	62 (18.9)	267 (81.1)	<0.001	2.64	1.43 — 4.87	0.002
≤10 (%)	19 (4.7)	389 (95.3)				
Tumor location (upper/middle/lower)	17/31/33	180/294/182	0.026			
Surgical extent			<0.001			
LT+UCND	0	91				
TT+UCND	0	301				
TT+BCND	81	264				
PT-LNM (%)	69 (34.8)	129 (65.2)	<0.001	11.2	5.62 — 22.32	<0.001
PL-LNM (%)	28 (48.3)	30 (51.7)	<0.001	4.3	2.16 — 8.55	<0.001
Ipa-LNM (%)	71 (20.1)	279 (79.9)	<0.001	2.84	1.32 — 6.09	0.007
Total number of metastatic lymph nodes	8.0 ± 4.5	1.3 ± 1.9	<0.001			
>5 (%)	52 (61.9)	32 (38.1)	<0.001			
≤5 (%)	29 (4.4)	624 (95.6)				

Cpa-LN, contralateral paratracheal lymph node; F, female; M, male; LT, lobe thyroidectomy; TT, total thyroidectomy; UCND, unilateral central lymph node dissection; BCND, bilateral central lymph node dissection; PT-LNM, pretracheal lymph node metastasis; PL-LNM, prelaryngeal lymph node metastasis; Ipa-LNM, ipsilateral paratracheal lymph node metastasis.

The analyses of potential risk factors for more than 5 metastatic central lymph nodes were shown in **Table 5**. Gender, age, capsular invasion, the largest diameter of tumor, tumor location, pretracheal lymph node metastasis, prelaryngeal lymph node metastasis, ipsilateral paratracheal lymph node metastasis, and contralateral paratracheal lymph node metastasis were included in the multivariate analysis. The larger size of the largest diameter of tumor (> 1cm; OR = 3.3, 95%CI 1.6 – 6.83; p = 0.001; **Table 5**), pretracheal lymph node metastasis (OR = 5.91, 95%CI 2.73 – 12.77; p < 0.001; **Table 5**), prelaryngeal lymph node metastasis (OR = 3.74, 95%CI 1.73 – 8.1; p = 0.001; **Table 5**), ipsilateral paratracheal lymph node metastasis (OR = 12.22, 95%CI 3.43 – 43.48; p < 0.001; **Table 5**), and contralateral paratracheal lymph node metastasis (OR = 7.68, 95%CI 3.86 – 15.3; p < 0.001; **Table 5**) were confirmed to be risk factors.

**Table 5:** The risk factors for more than 5 metastatic central lymph nodes.

Variables	Univariate Analysis			Multivariate Analysis (G=253.87, P<0.001)		
	Total number of metastatic lymph nodes		P	OR	95%CI	P
	>5 (n=84)	≤5 (n=653)				
Gender (F/M)	50/34	481/172	0.007			
Age (years)	38.0 ± 11.4	44.5 ± 12.4	<0.001			
≥55	8	131	0.02			
<55	76	522				
Hypertension	7	68	0.553			
Diabetes	0	19	0.152			
Hyperthyroidism	4	19	0.321			
Hypothyroidism	2	9	0.362			
Hashimoto’s Thyroiditis	24	187	0.99			
Nodular goiter	45	341	0.815			
Multifocal tumors	11	71	0.542			
Capsular invasion	48	205	<0.001			
Largest tumor size (mm)	17.6 ± 8.3	11.2 ± 6.7	<0.001			
>10 (%)	68 (20.7)	261 (79.3)	<0.001	3.3	1.6 — 6.83	0.001
≤10 (%)	16 (3.9)	392 (96.1)				
Tumor location (upper/middle/lower)	17/33/34	180/292/181	0.02			
Surgical extent			<0.001			
LT+UCND	0	91				
TT+UCND	5	296				
TT+BCND	79	266				
PT-LNM (%)	73 (36.9)	125 (63.1)	<0.001	5.91	2.73 — 12.77	<0.001
PL-LNM (%)	30 (51.7)	28 (48.3)	<0.001	3.74	1.73 — 8.1	0.001
Ipa-LNM (%)	81 (23.1)	269 (76.9)	<0.001	12.22	3.43 — 43.48	<0.001
Cpa-LNM (%)	52 (64.2)	29 (35.8)	<0.001	7.68	3.86 — 15.3	<0.001

F, female; M, male; LT, lobe thyroidectomy; TT, total thyroidectomy; UCND, unilateral central lymph node dissection; BCND, bilateral central lymph node dissection; PT-LNM, pretracheal lymph node metastasis; PL-LNM, prelaryngeal lymph node metastasis; Ipa-LNM, ipsilateral paratracheal lymph node metastasis; Cpa-LNM, contralateral paratracheal lymph node metastasis.

It was detailed in **Table 6** that the correlations of different combinations of different statuses of prelaryngeal and pretracheal lymph nodes with ipsilateral paratracheal lymph node metastasis, contralateral paratracheal lymph node metastasis, and more than 5 metastatic central lymph nodes. The more the metastatic prelaryngeal and/or pretracheal lymph nodes was, the higher the incidences of ipsilateral paratracheal lymph node metastasis, contralateral paratracheal lymph node metastasis, and more than 5 metastatic central lymph nodes were. The incidences of ipsilateral and contralateral paratracheal lymph node metastasis were more than 58% and 35% in patients with prelaryngeal lymph node metastasis, respectively (**Table 6**). During patients with pretracheal lymph node metastasis, the incidences of ipsilateral and contralateral paratracheal lymph node metastasis were 74% and 25%, respectively (**Table 6**). When the number of pretracheal metastatic lymph nodes was more than one, the incidences of ipsilateral paratracheal lymph node metastasis, contralateral paratracheal lymph node metastasis, and more than 5 metastatic central lymph nodes were more than 85%, 37%, and 49%, respectively (**Table 6**). When there was no prelaryngeal and pretracheal lymph node metastasis, the incidence of ipsilateral paratracheal lymph node metastasis still reached up to 34.5%, but the incidence of contralateral paratracheal lymph node metastasis and more than 5 metastatic central lymph nodes were only 1.1% and 1.7% (**Table 6**). However, when patients simultaneously suffered from prelaryngeal and pretracheal lymph node metastasis, the incidences of ipsilateral paratracheal lymph node metastasis, contralateral paratracheal lymph node metastasis, and more than 5 metastatic central lymph nodes were 85.7%, 50%, and 35.7%, respectively (**Table 6**).

**Table 6:** The effect of different combinations of different statuses of prelaryngeal and pretracheal lymph nodes on paratracheal lymph node metastasis and more than 5 metastatic central lymph nodes.

Variables		Ipa-LNM			Cpa-LNM			Total number of metastatic lymph nodes		
		≥1	0	P	≥1	0	P	>5	≤5	P
PL-LNM (+)										
PT-LNM (-)	0	10 (58.8)	7 (41.2)	0.009	6 (35.3)	11 (64.7)	0.211	2 (11.8)	15 (88.2)	<0.001
PT-LNM (+)	1	12 (85.7)	2 (14.3)		7 (50)	7 (50)		5 (35.7)	9 (64.3)	
	≥2	25 (92.6)	2 (7.4)		15 (55.6)	12 (44.4)		23 (85.2)	4 (14.8)	
Total		47 (81.0)	11 (19.0)		28 (48.3)	30 (51.7)		30 (51.7)	28 (48.3)	
PL-LNM (-)										
PT-LNM (-)	0	180 (34.5)	342 (65.5)	<0.001	6 (1.1)	516 (98.9)	<0.001	9 (1.7)	513 (98.3)	<0.001
PT-LNM (+)	1	71 (74.0)	25 (26.0)		24 (25)	72 (75)		15 (15.6)	81 (84.4)	
	≥2	52 (85.2)	9 (14.8)		23 (37.7)	38 (62.3)		30 (49.2)	31 (50.8)	
Total		303 (44.6)	376 (55.4)	<0.001	53 (7.8)	626 (92.2)	<0.001	54 (8.0)	625 (92.0)	<0.001

Ipa-LNM ipsilateral paratracheal lymph node metastasis, Cpa-LNM contralateral paratracheal lymph node metastasis, PL-LNM prelaryngeal lymph node metastasis, PT-LNM pretracheal lymph node metastasis.

## Discussion

The present study indicated that the larger size of the largest diameter of tumor (> 1cm) was a risk factor for pretracheal lymph node metastasis, prelaryngeal lymph node metastasis, paratracheal lymph node metastasis, and more than 5 metastatic central lymph nodes, and that both prelaryngeal lymph node metastasis and pretracheal lymph node metastasis were risk factors for paratracheal lymph node metastasis and more than 5 metastatic central lymph nodes, that capsular invasion was a risk factor for prelaryngeal lymph node metastasis and pretracheal lymph node metastasis.

In the present study, the incidence of occult central lymph node metastasis was 54.1% in unilateral lobe cT1-2N0 PTC, which was consistent with previous studies (1–3, 6). The incidence of ipsilateral paratracheal lymph node metastasis was 47.5%, while the incidence of more than 5 metastatic central lymph nodes was 11.4%. In consideration of the non-negligible incidence of more than 5 metastatic central lymph nodes, the great gap between this incidence and the incidence of occult central lymph node metastasis, and the increasing likelihood of temporary morbidity for prophylactic central lymph node dissection (4, 13), a feasible and reliable method without increasing the likelihood of temporary morbidity to identify PTC with more than 5 metastatic central lymph nodes from unilateral lobe cT1-2N0 PTC was necessary.

During patients with PTC of which the largest diameter was more than 1 cm in this study, the incidences of central lymph node metastasis, pretracheal lymph node metastasis, prelaryngeal lymph node metastasis, ipsilateral paratracheal lymph node metastasis, contralateral paratracheal lymph node metastasis, and more than 5 metastatic central lymph nodes were 67.8%, 38.3%, 12.2%, 59.9%, 18.9%, and 20.7%, respectively. The larger diameter increased the risk of central lymph node metastasis, which was also revealed by previous studies (8, 14, 15). The larger diameter means stronger invasiveness and/or a longer developmental time, which might increase the risk of lymph node metastasis (3, 14, 16).

Previous studies have suggested the predictive value of prelaryngeal lymph node metastasis to contralateral paratracheal lymph node metastasis, central lymph node metastasis, and lateral lymph node metastasis (3, 17, 18). The present study got similar results. One reason for not performing prophylactic central lymph node dissection for cT1-2N0 PTC in 2015 ATA Guidelines is that it increases the risk for temporary morbidity. Prelaryngeal lymph node dissection and pretracheal lymph node dissection could avoid this complication. What’s more, they could be performed before thyroidectomy and then the tissues were checked by intraoperative frozen pathology, which could avoid the increase in surgery time. And the evidence that prelaryngeal lymph node metastasis was a poor prognostic factor in laryngeal and hypopharyngeal cancers gave enlightenment that it might affect the prognosis of PTC (19, 20). Based on those, the significance of prelaryngeal and/or pretracheal lymph node metastasis in PTC was studied (3, 10, 17).

A meta-analysis suggested that the sensitivities of prelaryngeal lymph node metastasis to predict central lymph node metastasis, contralateral central lymph node metastasis, and lateral lymph node metastasis were 32%, 46%, and 52%, respectively (21). A reason for the unsatisfactory sensitivities might be the not-high existing rate of prelaryngeal lymph nodes, which ranged from 23% to 38% (7, 8, 22, 23). The number of prelaryngeal lymph nodes ranged from 0 to 2, with a median number was 0 (24, 25). While the number of pretracheal lymph nodes varied from 0 to 35, with an average number was 12.4 ( ± 8.2) (24, 25). Several studies indicated that the sensitivities of prelaryngeal and/or pretracheal lymph node metastasis to predict ipsilateral central lymph node metastasis and contralateral central lymph node metastasis varied from 38.7% to 66% and from 32.3% to 56.1%, respectively (6, 11, 26, 27). In consideration of the difference in the number between prelaryngeal lymph nodes and pretracheal lymph nodes, and the lack of study of the significance of a different number of metastatic lymph nodes, the predictive values of a combination of prelaryngeal lymph node metastasis and a different number of metastatic pretracheal lymph nodes were studied in the study.

Due to the abundant intersecting lymph vessels and complex lymphatic drainage of the thyroid, there is no precise sentinel lymph node for thyroid carcinoma (10). As a part of perithyroidal lymph node metastasis, prelaryngeal and/or pretracheal lymph node metastasis was a reflection of ipsilateral central lymph node metastasis and a transfer station of contralateral central lymph node metastasis. In the present study, when patients suffered from 0 metastatic prelaryngeal lymph nodes and more than one metastatic pretracheal lymph node, approximately half of them were found more than 5 metastatic central lymph nodes, the predictive value of which was close to that of prelaryngeal lymph node metastasis. During patients with more than two metastatic prelaryngeal and/or pretracheal lymph nodes, 71.2% were found more than 5 metastatic central lymph nodes. When the prelaryngeal lymph nodes metastasis and more than one metastatic pretracheal lymph node simultaneously occurred, the incidence of more than 5 metastatic central lymph nodes was 85.2%, and it even exceeded 90% in patients with the largest diameter more than 1 cm, the predictive value of which was far higher than that of prelaryngeal lymph node metastasis. For these patients, total thyroidectomy and prophylactic central lymph node dissection might be necessary.

According to the American Joint Committee on Cancer eighth edition cancer staging manual, the minor extrathyroidal extension detected only on histologic examination was removed from the definition of T3 disease (28, 29). While previous studies found that capsular invasion was a risk factor for prelaryngeal lymph node metastasis and contralateral paratracheal lymph node metastasis (7, 30). During the present study, capsular invasion was confirmed to increase the risk of prelaryngeal lymph node metastasis and pretracheal lymph node metastasis, but it did not convert to a risk factor for paratracheal lymph node metastasis and more than 5 metastatic central lymph nodes. The phenomenon might attribute to the fact that prelaryngeal lymph node metastasis and pretracheal lymph node metastasis included its’ influence. Several studies also suggested that minor extrathyroidal extension was far less of an independent prognostic risk factor (31–33).

Male was found to be a risk factor for prelaryngeal lymph node metastasis and/or central lymph node metastasis for PTC in several studies (23, 34, 35). A similar result was obtained in the present study. Although female was more prone to suffer from PTC, the male might be an invasion factor. The pretracheal lymph node metastasis occurred more in the younger in the present study, which was consistent with the previous study (3). It might be a result of the guess that the carcinoma was more aggressive when it occurred in younger patients. The non-upper lesion was more closed to the pretracheal lymph node, so it might be more likely to lead to pretracheal lymph node metastasis. The present study suggested that unifocal lesion increased the risk of pretracheal lymph node metastasis, which was contrary to the previous studies (3, 23, 36). One reason for the phenomenon might be that previous studies included bilateral lobe lesions and/or isthmic lesion. Another reason might be that multifocal lesions was more actively and earlier treated in view of the higher invasive of which, so there was less chance for metastasis.

There are several limitations to the study. First, it is limited by its retrospective nature. Second, the study was conducted in a single center. Third, contralateral paratracheal lymph node dissection was not routinely performed, which was consistent with real-world clinical practice, while which might lead to data biases. Fourth, because of the lack of availability, molecular markers (such as BRAF V600E or TERT promoter mutations) were not included in the study.

## Conclusion

In summary, patients with the larger size of the largest diameter of tumor (> 1cm), pretracheal lymph node metastasis, prelaryngeal lymph node metastasis, ipsilateral paratracheal lymph node metastasis, and contralateral paratracheal lymph node metastasis might be more likely to suffer from more than 5 metastatic central lymph nodes (intermediate-risk). And prelaryngeal and pretracheal lymph node metastasis could help to identify PTC with more than 5 metastatic central lymph nodes from unilateral lobe cT1-2N0 PTC. Based on the incidence of more than 5 metastatic central lymph nodes, total thyroidectomy and contralateral paratracheal lymph node dissection should be taken into consideration and ipsilateral central lymph node dissection might be necessary when the number of metastatic prelaryngeal and/or pretracheal lymph node was more than one; total thyroidectomy and ipsilateral central lymph node dissection should be performed and contralateral paratracheal lymph node dissection might be also necessary, especially for PTC with the largest diameter more than 1 cm, when more than two metastatic prelaryngeal and/or pretracheal lymph nodes occurred. The larger the number of metastatic prelaryngeal and/or pretracheal lymph nodes was, the more likely paratracheal lymph node metastasis was to occur. More studies are necessary to validate the results of the retrospective study.

## Data availability statement

The raw data supporting the conclusions of this article will be made available by the authors, without undue reservation.

## Ethics statement

The studies involving human participants were reviewed and approved by Medical Ethics Committee of The Third People’s Hospital of Chengdu. The patients/participants provided their written informed consent to participate in this study.

## Author contributions

Conceptualization: BW, C-RZ, YF, HL, X-MY and JW. Methodology: BW, C-RZ and YF. Software: BW and YF. Validation: C-RZ, HL and X-MY. Formal analysis: BW and C-RZ. Data curation: BW and C-RZ. Writing—original draft preparation: BW and C-RZ. Writing—review and editing: YF and HL. Visualization: X-MY. Supervision: X-MY and JW. Project administration: X-MY and JW. Funding acquisition: BW and JW. All authors have read and agreed to the published version of the manuscript. All authors contributed to the article and approved the submitted version.
